# Smartphone GPS-Based Exposure to Greenspace and Walkability and Accelerometer-Assessed Physical Activity During Pregnancy and Early Postpartum—Evidence from the MADRES Cohort

**DOI:** 10.1007/s11524-024-00903-6

**Published:** 2024-08-15

**Authors:** Li Yi, Rima Habre, Tyler B. Mason, Yan Xu, Jane Cabison, Marisela Rosales, Daniel Chu, Thomas A. Chavez, Mark Johnson, Sandrah P. Eckel, Theresa M. Bastain, Carrie V. Breton, John P. Wilson, Genevieve F. Dunton

**Affiliations:** 1 Spatial Sciences Institute, University of Southern California, Los Angeles, CA USA; 2 Department of Population and Public Health Sciences, University of Southern California, Los Angeles, CA USA; 3 Departments of Civil & Environmental Engineering, Computer Science, and Sociology, University of Southern California, Los Angeles, CA USA; 4 Department of Psychology, University of Southern California, Los Angeles, CA USA

**Keywords:** Pregnancy, Physical activity, Built environment, Green space, Walkability, GPS, Accelerometer

## Abstract

A growing number of studies have associated walkability and greenspace exposure with greater physical activity (PA) in women during pregnancy. However, most studies have focused on examining women’s residential environments and neglected exposure in locations outside the home neighborhood. Using 350 person-days (*N* = 55 participants) of smartphone global positioning system (GPS) location and accelerometer data collected during the first and third trimesters and 4–6 months postpartum from 55 Hispanic pregnant women from the Maternal and Developmental Risks from Environmental and Social Stressors (MADRES) study, we examined the day-level effect of women’s exposure to walkability and greenspace on their PA outcomes during pregnancy and in the early postpartum period. Moderate-to-vigorous physical activity [MVPA] minutes per day was assessed using accelerometers. Walkability and greenspace were measured using geographic information systems (GIS) within women’s daily activity spaces (i.e., places visited and routes taken) recorded using a smartphone GPS and weighted by time spent. We used a generalized linear mixed-effects model to estimate the effects of daily GPS-derived environmental exposures on day-level MVPA minutes. Results showed that women engaged in 23% more MVPA minutes on days when they had some versus no exposure to parks and open spaces in activity spaces (*b* = 1.23; 95%CI: 1.02–1.48). In addition, protective effects of daily greenspace and walkability exposure on MVPA were stronger in the first and third trimesters, among first-time mothers, and among women who had high pre-pregnancy body mass index (BMI) and lived in least-safe neighborhoods. Our results suggest that daily greenspace and walkability exposure are important for women’s PA and associated health outcomes during pregnancy and early postpartum.

**Supplementary Information:**

The online version contains supplementary material available at 10.1007/s11524-024-00903-6.

## Introduction

Physical inactivity during pregnancy is associated with short- and long-term maternal and infant health outcomes pre- and post-partum, such as increased gestational weight gain, cardiometabolic diseases, and mental health concerns [1–3]. Evidence suggests that Hispanic women in the USA are at disproportionately high risk for pregnancy-related obesity and negative cardiometabolic health outcomes, and overall are less likely to adhere to physical activity (PA) guidelines for pregnant women than the non-Hispanic white population [4]. Understanding the factors that influence Hispanic women's PA behaviors is a critical step toward reducing the disproportionate obesity risk and health consequences faced by this group.

A large body of research has shown that higher greenspace exposure and walkable environments are associated with higher PA levels in adults [5, 6]. According to the socio-ecological model of PA [7], green spaces could influence PA by directly providing an aesthetically pleasing or shaded environment for recreational PA such as jogging or sports activities [8, 9]; while a walkable environment could provide both destinations within walking distance (e.g., grocery stores) and pedestrian-friendly infrastructure that promote walking to accomplish activities of daily living (e.g., grocery shopping) [10]. In terms of pregnant women, previous studies have associated higher levels of exposure to greenness, better access to public transportation, and walkability with higher levels of PA in women during pregnancy and postpartum [11–14]. In addition, several studies of Hispanic adults have found that the presence of sidewalks and access to parks and recreational facilities are associated with higher levels of PA [15, 16]. Nevertheless, there are no studies to date that have examined the association between exposure to walkability and greenspace and PA in Hispanic women during pregnancy. Moreover, most of the studies mentioned above refer to a single place of residence and a specific time point to assess exposure to greenspace and walkability for each participant (primarily derived from addresses reported on birth certificates or postpartum questionnaires). This approach does not capture women’s exposure at locations other than home (e.g., at work) and ignores residential mobility and dramatic variations in activities (e.g., grocery shopping, commuting to work, and visiting a park) during pregnancy and postpartum, which could have important impacts on PA [17]. As a result, the static, residence-based approach, in which exposure is measured at a single point in time, can lead to measurement error in exposure and thus bias in the results of the study. This issue is often specified as the uncertain geographic context problem (UGCoP) [18], which refers to the uncertainties of contextual exposure affecting health behaviors or outcomes.

To mitigate UGCoP, increasingly, studies on pregnant women are turning to Global Positioning System (GPS)-based exposure assessment approaches [17, 19–23]. Compared to residential-based approach, the GPS-based approach infers the environmental exposure of individuals within the participants’ daily activity spaces (i.e., places visited and routes taken). This reduces measurement errors in environmental exposure and mitigates potential biases in the study results. Moreover, GPS-based exposures can be paired with accelerometer-measured PA data to examine associations between greenspace and walkability and PA outcomes at higher temporal resolution, such as daily or minute level [24]. Nevertheless, no study has investigated the association between GPS-based exposure to the built environment and greenspace and PA in pregnant women.

In this study, we paired smartphone-based GPS application (app) and objective accelerometers to examine the extent to which daily exposure to greenspace and walkability was associated with PA outcomes during the first and third trimesters of pregnancy and 4 to 6 months postpartum in a group of Hispanic, predominantly low-income women from the Maternal And Developmental Risks from Environmental and Social stressors (MADRES) study in Los Angeles, CA, USA. In addition, exposures to greenspace and walkability and PA outcomes may vary by temporal factors such as weekdays versus weekend days and pregnancy periods [17]. In addition, the associations between the two could also be influenced by maternal parity and pre-pregnancy body mass index (BMI) categories [25]. In addition, NSES and neighborhood crime could also modify the associations [26]. Therefore, we explored whether the association between daily exposure and PA differed by weekdays vs. weekends, pregnancy and postpartum periods, individual demographic characteristics, and neighborhood socioeconomic status (NSES).

## Methods

### Study Population

We used data from the Real-Time and Personal Sampling sub-study of the larger MADRES study, which used a multi-wave intensive longitudinal design. A total of 65 Hispanic, predominantly lower income mothers from the larger MADRES prospective cohort were recruited on a rolling basis between 2016 and 2018 in Los Angeles, CA, USA [27]. The inclusion criteria for the larger MADRES study included a) at least 18 years old with a singleton pregnancy and 2) at less than 30 weeks’ gestation at time of recruitment. The exclusion criteria included a) HIV positive; b) physical, mental, or cognitive disabilities that prevented participation; and c) currently incarcerated. Recruitment of Hispanic 65 women occurred on a rolling basis between 2016 and 2018 from one county hospital prenatal clinic (*n* = 16) and one non-profit community health clinic (*n* = 49). Additional eligibility criteria for this sub-study are described in further detail in O’Connor et al. [27]. This study was approved by the Institutional Review Board of the University of Southern California.

### Exposure Assessments

#### Location Information using Global Positioning Systems (GPS)

Study participants were provided with a Samsung MotoG phone with Android operating system (Google, Mountain View, CA, USA), which they carried for four days (two weekdays and two weekend days) during three separate waves (i.e., first and third trimesters and 4–6 months postpartum). During this time, high-resolution and encrypted geolocation data was collected at 10-s intervals via a pre-installed smartphone app (madresGPS app) developed by the MADRES researchers [17]. The 10-s epoch of GPS geolocation data was then processed and missing data was imputed using a custom algorithm with details described in Yi et al. (2022) [17]. We flagged days with < 6 h of GPS data (after imputation) as invalid and decided not to include them in subsequent analyses. This decision considers the diurnal patterns of missing data and allows us to preserve the quality of GPS data and maximize data retention for subsequent analyses, such as environmental exposure assessment.

#### Constructing Activity Spaces

**Fig. 1:**
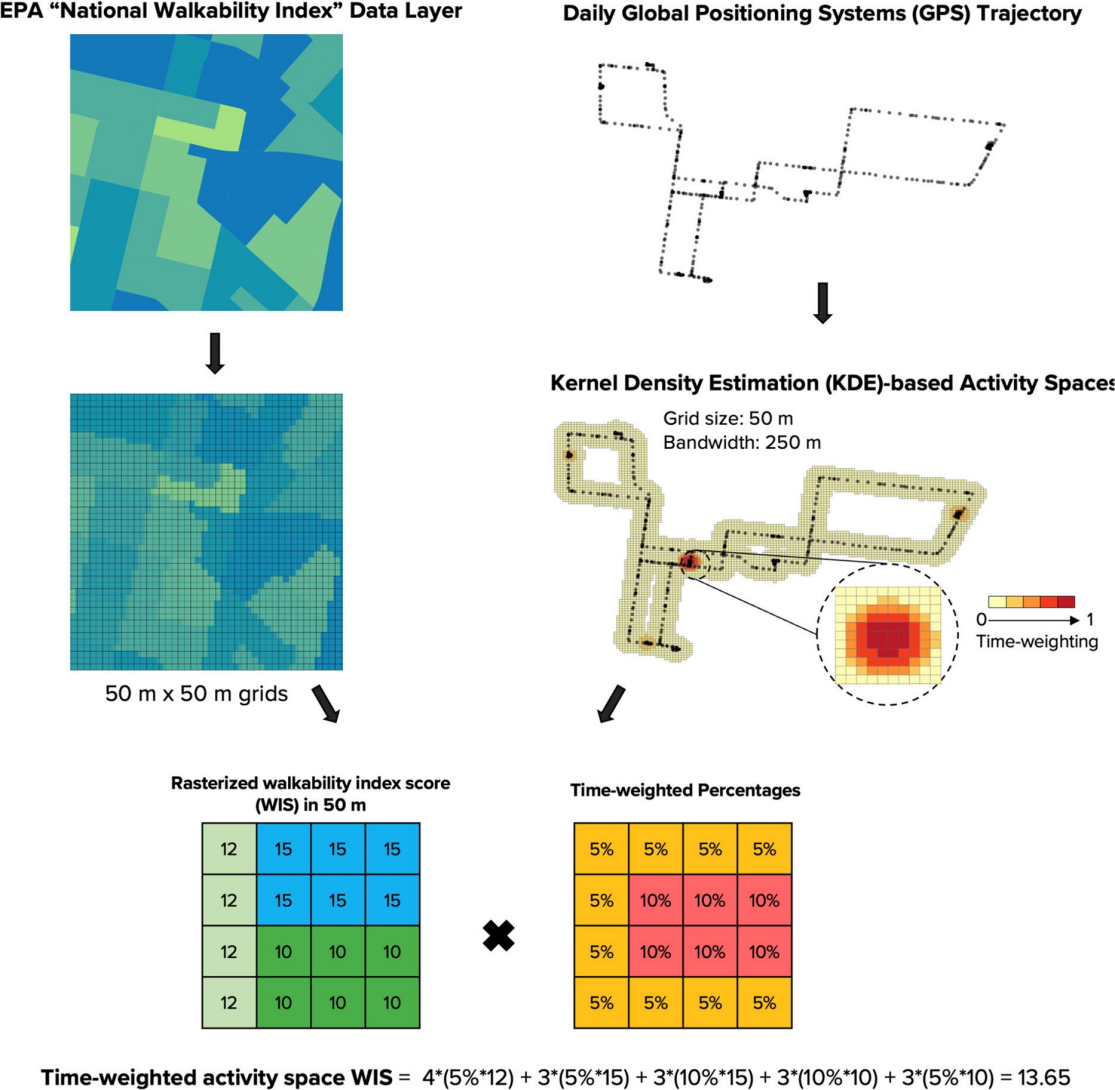
An illustration of KDE-based activity space exposure assessment approach

We constructed participants’ daily activity spaces in the first and third trimesters and 4–6 months postpartum by applying the kernel density estimation (KDE) spatial method [24]. As shown in Fig. 1, the KDE method generates time-weighted activity grids with 50 × 50 m cells with a 250-m bandwidth. Each cell was assigned a value based on the percentage of time spent within a given day as recorded by GPS trajectories. We chose 50 m to account for potential noise in smartphone GPS data. This is based on the results of a previous study in which we found that certain urban environments (e.g., under high-rise buildings, streets with heavy tree cover) affect the accuracy of smartphone GPS location data [17]. We chose a buffer size of 250 m, which corresponds to the depth of several city blocks in urban Los Angeles, CA, to account for both visual exposure (e.g., parks along streets) and perception (e.g., nearby gyms and recreational facilities in areas of high walkability) [19]. We performed all operations using ArcGIS Pro 10.7.1 (Esri, Redlands, CA, USA).

#### Derive Exposure Metrics

After constructing the daily activity spaces, we assessed participants’ exposure to neighborhood greenness, parks, and open space, as well as the walkability of these spaces. For this study, we chose four exposure metrics that have been commonly used in previous studies examining the relationship between the built environment and PA in adult population, especially pregnant women [11–14, 19, 28]. These included the street-level greenspace coverage, the distance to the nearest park entrance (m), exposure to parks and open spaces on public lands (e.g., grass, orchards, riverside, and beach), and the walkability index score (from 1 to 20; a higher value indicates higher walkability). The spatial operations used to calculate the four exposure metrics, along with the data source, layer name, and illustration are summarized in Table 1 and documented in detail in a prequel study [19]. In brief, we gridded spatial datasets (e.g., walkability index score values at the census tract level) into 50 m × 50 m grid cells and multiplied the values of each cell (e.g., walkability index score value = 10) by the value (time weighting, e.g., 0.12 represents 12% of time spent in this grid within a day) of the corresponding cell of the daily KDE grids. We then determined the daily exposure values as weighted sum by adding up the values across all grid cells (see Fig. 1 for an illustration), except for daily parks and open space exposure, which we classified as a binary exposure (yes vs. no) variable based on its distributions.

**Table 1:** List of daily greenspace and walkability measures, along with the corresponding data source, layer name and description, and the steps of spatial operations to derive the exposure metric

Measures	Data Source	Data Layer Name, Illustration, and Description	Steps of Spatial Operations
Street-level greenspace coverage (%)	2008-2013 EPA EnviroAtlas Community Data	**“Percent green space along walkable roads”** This data layer measures the percentage of green space areas within 25m buffers of street segments. According to the documentation, green space areas were derived by combining areas of multiple land cover classes including water, trees and forest, grass and herbaceous cover, shrubs, agriculture, orchards, and woody and emergent wetlands. Sidewalk areas were derived by buffering r NAVTEQ (Chicago, IL, USA) street segments with a speed limit less than 55 mph (potentially walkable roads) by a width of 25 m on each side. More details on this data can be found at EPA Enviroatlas website.	To derive the daily metric for street-level greenspace coverage (%): 1. The street network segments data layer was buffered by 25 m on each side and gridded into a 50 m × 50 m grid to match our daily kernel density estimation (KDE) surfaces 2. The value of each grid (i.e., % of time spent) in the daily KDE surface was multiplied by the value of each grid (e.g., 51% coverage) in the data layer 3. Derived the daily value for street-level greenspace coverage (%) by summing the multiplied values of all grids
Proximity to the nearest park (m)	2011 EPA EnviroAtlas Community Data	**“Estimated walking distance to a park entrance (m)”** This measure was derived by delineating approximate walking areas from a park entrance at any given location within the EnviroAtlas community boundary (i.e., Los Angeles County). More details on this data can be found at EPA Enviroatlas website.	To derive the daily proximity to the nearest park (m) metric: 1. The data layer was into a 50 m × 50 m grid 2. The value of each grid (i.e., % of time spent) in the daily KDE surface was multiplied by the value of each grid (e.g., 3,500 m) in the data layer 3. Derived the daily value for proximity to the nearest park (m) by summing the multiplied values of all grids
Parks and open space exposure (yes/no)	2021 California Protected Areas Database	**“California Protected Areas Database”** California protected areas include: 1) National/state/regional parks, forests, preserves, and wildlife areas; 2) large and small urban parks that are mainly open space (as opposed to recreational facility structures); 3) land trust preserves; and 4) Special district open space lands (watershed, recreation, etc.) and other types of open space. More details on this data can be found at CPAD Website.	To derive the daily parks and open space exposure metric: 1. The data layer was into a 50 m × 50 m grid 2. The value of each grid (i.e., % of time spent) in the daily KDE surface was multiplied by the value of each grid (i.e., 1 if the grid is a park, 0 if not) in the data layer 3. Derived the daily parks and open space exposure from whether there was parks and open space exposure (>0 mi^2^) in a daily KDE surface (Yes vs. No)
Walkability index score (range from 1 to 20)	2021 EPA Smart Location Database	**“National Walkability Index”** National Walkability Index is a composite index score combining household and employment density, street intersection density, and distance to nearest transit stops. This measure represents different built environment characteristics that are known to be supportive of walking. The index scores range from 1 to 20, with higher value represents better walkability. More details on this data can be found at EPA website.	To derive the daily walkability index score metric: 1. we first gridded the data layer into a 50 m × 50 m grid to match our daily KDE surfaces 2. The value of each grid (i.e., % of time spent) in the daily KDE surface was multiplied by the value of each grid (e.g., a score of 15) in the gridded data layer 3. Derived the daily value for walkability index score (from 1 to 20) by summing the multiplied values of all grids

*KDE*, kernel density estimation

### PA Outcome

We used the wGT3X-BT ActiGraph accelerometer to measure women’s PA during pregnancy and early postpartum. The women were instructed to wear the accelerometer for 4 consecutive days (2 weekdays and 2 weekend days) in each study period. The accelerometer was attached to an adjustable belt and worn on the right hip at all times except when sleeping, bathing/showering, or swimming [27]. Body movement data were recorded by the accelerometer in activity count units for each 10-s epoch. Non-wear periods were defined as > 60 continuous minutes without an activity count and non-valid days were defined as < 10 h of wear [27], both of which were removed from the analyses. To be consistent with national surveillance data, moderate-to-vigorous physical activity (MVPA), a PA intensity type that reduces the risk of many adverse health outcomes [29], was identified as a Freedson prediction equation above four metabolic equivalents of task (METs = 1.439008 + 0.000795 × counts·min^−1^) [29]. The day-level MVPA outcome variable was created by summing the total number of 10-s MVPA epochs within an observation day and converting to a unit of minutes.

### Covariates

We examined the following individual-level covariates, which were a priori determined to be potential confounders as they might be associated with PA outcomes and/or correlated with environmental exposures: age at enrollment, education (some college or graduate degrees/high school diploma or less), maternal parity (first born/second or greater birth), pre-pregnancy BMI categories (normal/overweight/obese), and self-reported employment status (yes/no; time varying, collected by questionnaire during first and third trimesters and 6 months postpartum). We controlled for area-level SES using deprivation index score (on a scale of 1–10, where 1 = least deprived) from the Neighborhood Atlas [30]. Scores were assigned to participants’ residences based on the 2010 Census block group boundary in which their neighborhoods were located. We did not adjust for individual household income because our participants were primarily a low-income group with little variation and therefore unlikely to influence the relationships between environmental exposure and PA outcomes. More importantly, we focused on within-person associations (over time) between daily greenspace and walkability exposures and day-level MVPA minutes, which would adjust for person-level differences as random effects in our mixed-effects models. In addition, we adjusted participants’ self-reported neighborhood cohesion and safety score (on a scale of 1–5, with 1 representing the least safe and least cohesive) on the questionnaires for the second trimester (chosen to represent pregnancy) and 6 months postpartum [31]. We also adjusted temporal factors including weekend versus weekday (1 = weekend), daily average temperature in degrees Celsius, and study period (the first and third trimesters and 4–6 months postpartum). Lastly, we included daily accelerometer wear time to adjust for individual device-wearing behaviors.

### Statistical Analysis

First, we examined Spearman’s rank correlations between all environmental exposures. To account for the interdependence of the nested data structure in the current study (level 1 days nested with level 2 participants), we used generalized linear mixed-effects models (GLMMs) with random intercepts at the participant level to test participant- and day-level effects of four daily GPS-derived environmental exposure metrics on day-level MVPA minutes. To test the necessity for a multilevel model (i.e., clustering of day-level MVPA outcomes within participants), we estimated the intraclass correlation coefficient (ICC) using GLMM models with only random intercepts (no covariates). The results showed that 41% (ICC = 0.41) of the variation in day-level MVPA minutes was between participants and 59% was within participants, which justifies the use of mixed models.

All models included all four exposure metrics (i.e., % greenspace along walkable routes, distance to the nearest park entrance, exposure to parks and open space, and walkability index score) as independent variables in the same model. All measures were centered on the person-mean to disentangle between-subject (participant-level) and within-subject (day-level) effects. In addition, the day-level MVPA outcome variable was logarithmically transformed to ensure normality, as the variable had a right-skewed distribution.

#### Effect Modification

To explore potential effect modification of pregnancy and postpartum periods (first trimester/third trimester/4–6 months postpartum), maternal parity (first born/second or greater birth), pre-pregnancy BMI (normal/overweight/obese), and neighborhood cohesion and safety score (on a scale of 1–10) on the daily associations between exposures to greenspace and walkability and PA outcomes, we fitted GLMM models with a multiplicative interaction term between each GPS-based environmental exposure metric and the effect modifier. For each significant interaction (*p* < 0.05), we estimated the predicted trajectories for MVPA min/d associated with each exposure metric at levels of the modifier (all categories if categorical; − 1SD, mean, + 1SD if continuous). We also performed simple slope analyses to estimate the predicted slope at levels of the modifier.

#### Sensitivity Analyses

We performed two sensitivity analyses to explore the robustness of our findings and to mitigate UGCoP, per the recommendation of a previous study [32]. First, we ran the models using GPS-based exposure metrics derived using smaller buffer sizes for KDE activity space method (100 m). Second, we ran the models using GPS-based exposure metrics derived using the daily path area activity space method, which captures participants’ exposure along daily paths instead of KDE-identified activity locations [24]. To construct the daily path area, we connected successive GPS points into lines based on timestamps (i.e., routes) and then buffered them with a 250-m radius. The buffer size of 250 m was chosen to match the KDE method and to facilitate the comparison of exposure metrics between the two methods. Third, we compared the GPS-based exposure metrics with the residential neighborhood-based metrics using the same four metrics derived within residential area network buffers (800 m and 1600 m). We chose these numbers because they have been commonly used in previous studies and correspond to a 5–10 min and 15–20 min walk, respectively [32]. All analyses were performed in R, version 4.2.1. (R Core Team, Vienna, Austria). As the outcome variable was log transformed, exponentiated effect estimates were reported for all models, which were interpreted on a multiplicative scale.

## Results

A total of 651 accelerometer-measured days of PA were recorded by 62 women. Among these, we removed 210 non-valid days with < 10 h of wear time. Examination of predictors of non-valid days revealed that weekend days were more likely to be invalid days; all other covariates had no effect on predicting valid or invalid days. In addition, 94 days of data collection were removed due to missing daily exposure measurement data due to no or low-quality GPS data. This resulted in a final analysis sample of 350 person-days that were valid accelerometer-measured days and with matched GPS exposure metrics derived (*N* = 55 participants). Our study participants were on average 29.0 (6.1) years old at baseline, all Hispanic, more than one-third (35%) had a college degree or more, and more than one-third were employed (36%). At baseline, 30% were pregnant with their first child and slightly less than three-quarters (73%) met criteria for overweight or obesity according to their pre-pregnancy BMI.

In terms of greenspace exposure, women on average were exposed to daily activity spaces with walkable roads that had 23.3% (8.3) green space coverage, which was lower than the LA County average of 32.4%. In terms of access to parks and open spaces, women’s average distance from any point in the daily activity spaces to the nearest park entrance was 790.8 m (398.5; equivalent to a 15–20-min walk). In addition, women were exposed to parks and open space in their activity spaces on about three-quarters of the observation days (73.4%; 257 out of 350 days). Finally, in terms of walkability, the mean walkability index score was 15.0 (1.7), 1.5 scales higher than the LA County average. As for PA outcomes, women on average were engaged in 30.7 (22.4) MVPA min/d across three study periods. The full descriptive statistics can be found in Table 2.

**Table 2:** Descriptive statistics for physical activity outcomes, GPS-derived exposure to greenspace and walkability metrics, and covariates of study participants (*N* = 55 participants,* N* = 350 person-days)

	Overall (*N* = 55 participants, *N* = 350 person-days)
Variable	Mean (SD) or *n* (%)
Outcome	
Day-level MVPA minutes	30.73 (22.45)
Exposure metrics	
% green space along walkable roads	23.30 (8.27)
Days with parks and open space exposure (yes)	257 (73.4%)
Distance to the nearest park entrance (in m)	790.80 (398.47)
Walkability index score (range from 1 to 20)	15.01 (1.68)
Covariates	
Age	29.04 (6.14)
Education	
High school or less	36 (65.45%)
Some college/graduate	19 (34.55%)
Parity	
Second or greater birth	39 (70.91%)
First-born	16 (29.09%)
Pre-pregnancy BMI categories	
Normal	15 (27.27%)
Overweight	19 (34.50%)
Obesity	21 (38.20%)
Employment status at enrollment	
Unemployed	30 (63.83%)
Employed	17 (36.17%)
Household income at enrollment	
Less than $15,000	9 (16.40%)
$15,000 to $29,999	19 (34.50%)
$30,000 to $49,999	12 (21.80%)
$ 50,000 or more	2 (3.64%)
Missing	13 (23.6%)
Neighborhood cohesion and safety score (range from 1 to 5)	3.02 (0.69)
Neighborhood deprivation index (range from 1 to 10)	6.34 (1.78)
Total person valid days	7.93 (3.51)
Daily accelerometer wearing hours	13.79 (2.63)
Person-days by study visit	
First trimester	133 (38.00%)
Third trimester	106 (30.30%)
4–6 months postpartum	111 (31.70%)
Type of day	
Weekday	191 (54.57%)
Weekend	159 (45.43%)
Average daily temperature (°C)	19.92 (4.40)

*GPS*, Global Positioning Systems, *KDE,* kernel density estimation

GLMM results examining within-subject (day-level) associations between greenspace and walkability exposure metrics and women’s day-level MVPA outcomes are presented in Table 3. After controlling for covariates, we found (see Table 3) on days when women were exposed to any parks and open space in activity spaces, they engaged in 21% more MVPA min/d (*b* = 1.21; 95%CI: 1.01–1.46). No associations were found between other exposure metrics and women’s MVPA outcomes at the day level. The results were almost the same (*b* = 1.22; 95%CI: 1.02–1.47) in the sensitivity analyses via 250-m daily path area model (see Table S1), but no longer significant in sensitivity analyses that applied exposure metrics derived using the 100-m KDE metrics (see Table S1). We found no associations between the exposure metrics derived using 800-m and 1600-m residential network buffer methods and the MVPA results at day level.

**Table 3:** Between- and within-subject effects of GPS-based greenspace and walkability exposures derived using the 250 m KDE method on daily MVPA outcomes

	Daily MVPA minutes(log-transformed)
	*250-m KDE model*
*Predictors*	*Estimates (95%CI)*^*1*^
% green space along walkable roads (BS)	1.01 (0.99–1.02)
Distance to the nearest park entrance (BS)	1.01 (0.98–1.04)
Walkability index score (BS)	1.05 (0.98–1.13)
Daily parks and open space exposure (BS)	1.69 * (1.02–2.82)
% green space along walkable roads (WS)	0.99 (0.9 –1.01)
Distance to the nearest park entrance (WS)	0.99 (0.94–1.04)
Walkability index score (WS)	1.01 (0.88–1.14)
Daily parks and open space exposure (WS)^2^	1.23 * (1.02–1.48)
Maternal age	0.99 (0.97–1.02)
Education: some college/graduate	0.66 ** (0.50–0.85)
Parity: first-born	1.02 (0.77–1.36)
Employment status: employed	1.03 (0.81–1.30)
Pre-pregnancy BMI category: overweight	0.93 (0.70–1.23)
Pre-pregnancy BMI category: obesity	0.71 * (0.54–0.93)
Parity: first-born	1.02 (0.77–1.36)
Average daily temperature (°C)	1.02 (1.00–1.04)
Type of day: weekend	0.81 ** (0.72–0.92)
The third trimester day	0.99 (0.78–1.26)
4–6 months postpartum day	0.90 (0.70–1.16)
Neighborhood cohesion and safety score (range from 1 to 5)	1.00 (0.88–1.15)
Neighborhood deprivation index (range from 1 to 10)	1.03 (0.96–1.10)
Daily accelerometry wearing hours	1.07 *** (1.04–1.11)

* p < 0.05, ** p < 0.01, *** p < 0.001

*KDE*, kernel density estimation; *BS*, between-subject effects; *WS*, within-subject (day-level) effects; *BMI*, body mass index

^1^Exponentiated effect estimates interpreting on a multiplicative scale were reported for all models

^2^The binary variable was not person-mean centered for the ease of interpretation. The person-mean centered versions of the variable were also tested, and results remained invariant

**Fig. 2:**
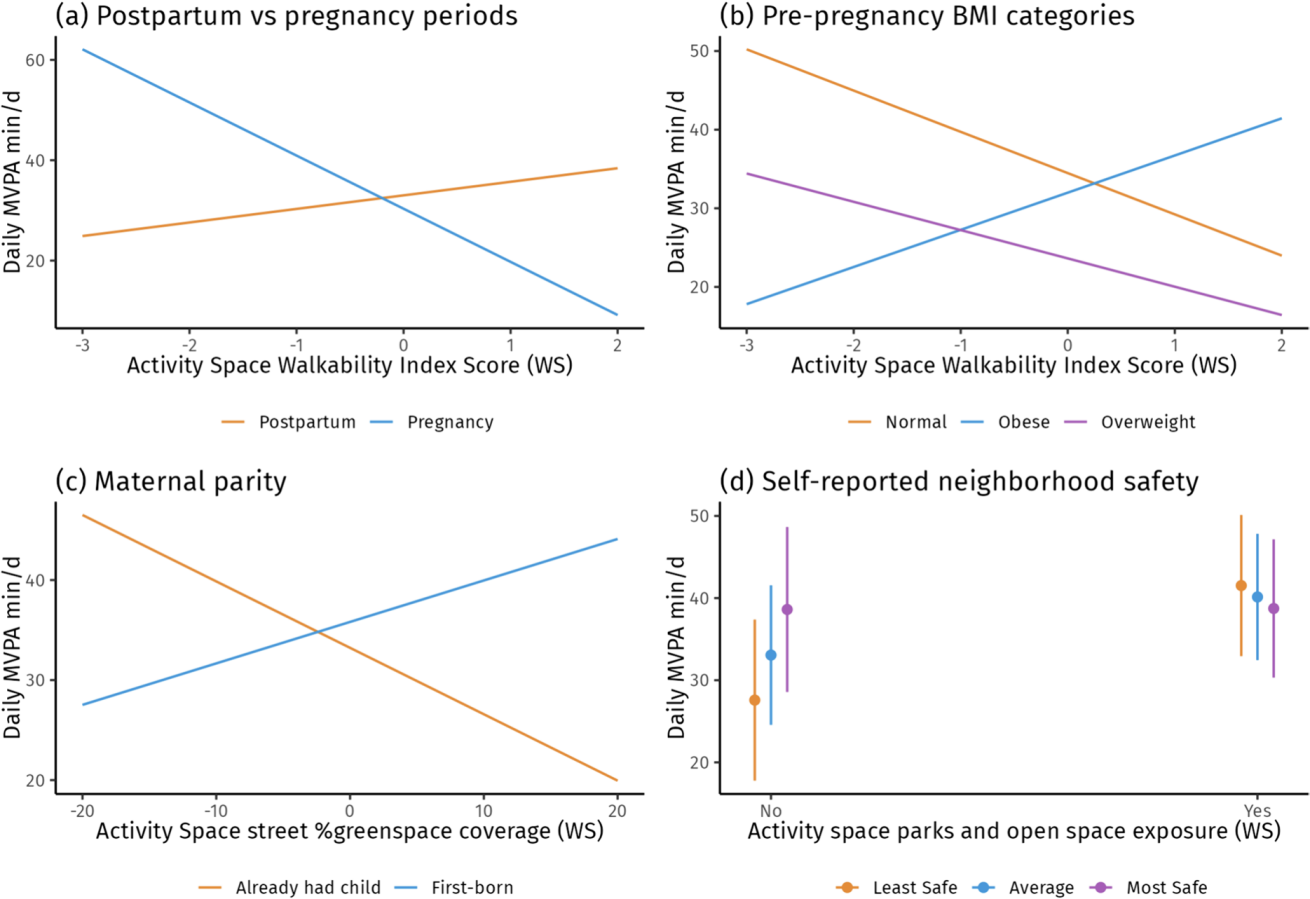
The significant day-level interactions between GPS-based greenspace and walkability exposure metrics and a list of temporal factors, individual health factors, and neighborhood characteristics in predicting MVPA min/day*.* KDE, kernel density estimation; WS, within-subject (day-level) effects; BMI, body mass index. Self-reported neighborhood safety thresholds based on neighborhood cohesion and safety score (range 1–5): < 2.8 = least safe; 2.8–3.2 = average; > 3.2 most safe

The day-level effect of walkability exposure on MVPA minutes differed by postpartum and pregnancy periods (Fig. 2a), although the simple slope analysis yielded non-significant results. We also found the effect differed by pre-pregnancy BMI categories (see Fig. 2b); for women with obesity, when they were exposed to a daily activity space with higher than usual walkability, they engaged in more MVPA min/d (*b* = 1.26; 95%CI: 1.02–1.56 for obesity group). Additionally, regarding effect modification by maternal parity (see Fig. 2c), for women who had their first-born child, on days when they were exposed to activity spaces with higher greenspace coverage along streets than usual, they engaged in more MVPA min/d (*b* = 1.02; 95%CI: 0.99–1.06 for first-time mothers). Lastly, in terms of neighborhood covariates, we observed evidence of effect modification by self-reported neighborhood safety for daily park and open-space exposure (Fig. 2d). Among women who reported their neighborhood less safe (− 1SD), on days when they were exposed to any parks and open space than usual, they engaged in more MVPA min/d (*b* = 1.57; 95%CI: 1.2–2.04 for -1SD neighborhood safety score). The results of simple slope analyses for all statistically significant interaction terms are available in Table S2.

## Discussion

In this study, conducted in urban Los Angeles using 350 person-days of high-resolution smartphone location and accelerometer data collected from a sample of 55 Hispanic, low-income pregnant women, we found that any daily exposure to parks and open space in women’s daily activity spaces was associated with higher day-level MVPA. The results were robust to the different spatial methods used to construct activity spaces, and no associations were found when the same associations were examined using exposure metrics derived in residential neighborhoods. Associations between daily exposure to greenspace and walkability and day-level MVPA differed by parity, pregnancy vs. postpartum periods, pre-pregnancy BMI, and self-reported neighborhood safety.

Our findings on the protective associations between daily parks and open space exposure and MVPA levels in pregnant women complement previous population-based studies that focused on differences in exposure between pregnant women and its effects on their PA outcomes and found mixed results [11–14, 33]. For example, a New Zealand study found that exposure to residential green space was not associated with PA during pregnancy [12], while a Norwegian study reported that women who lived in neighborhoods with good access to recreational areas had more MVPA than women who had limited access [14]. This discrepancy could be due to the static, residential approach to assessing exposure to green space used in both studies, which may not capture the exposure of women in outside residential neighborhoods and therefore biasing the study results toward the null [24]. This is supported by the results of our sensitivity analyses, in which we found that exposure to parks and open space in residential areas, derived using 800-m and 1600-m network buffers, was not associated with women’s day-level MVPA during pregnancy and in the early postpartum period.

We found evidence of effect modification by several factors for the associations between greenspace and walkability exposure and women’s PA during pregnancy. First, we found a positive association between day-level activity space walkability exposure and women’s PA during pregnancy but not in early postpartum, which may be due to recovery, rest, and infant care responsibilities in early postpartum [34]. We also found that the positive effect of walkability on day-level MVPA only occurred in women who had given birth to their first child. It could be that women who were not primiparous had more caregiving responsibilities than their counterparts, which reduced activity [35]. Moreover, our results indicated daily exposures to activity spaces with higher walkability than usual were associated with an increase in MVPA among women with obesity, whereas the direction was reversed in other weight groups. Women without obesity may be more likely to be members of sports clubs such as gyms or fitness clubs, or they may achieve a large proportion of their daily physical activity through housework and occupational activities; as a result, the activity space walkability may have less influence on their MVPA. In contrast, given the linear relationship between BMI and weight stigma found in previous research [36], individuals with obesity may be most likely to report weight stigma, either actual or expectations, in gyms or fitness clubs. Therefore, obese women may prefer outdoor walks, which can be facilitated by a walkable environment. Furthermore, women’s self-reported neighborhood safety modified the association between greenspace exposure and MVPA. The association was only significant for women who reported living in the least-safe neighborhoods. Previous studies have found a negative association between neighborhood safety and PA in pregnant women [37, 38]. Therefore, it is possible that exposure to greenspace and walkable environments near non-home locations and along travel routes may create additional PA opportunities for those who were inactive due to greater safety concerns in their own neighborhoods.

Our study also has a few limitations. First, the GPS data had some missingness that could potentially bias our exposure measurements. In addition to this, we only collected 4-day GPS data on two weekdays and two weekend days in each study period. Therefore, the time-activity and mobility patterns identified from our samples may not capture some infrequent activities that are more likely to occur weekly or on other days of the week, such as grocery shopping. Second, we were not able to distinguish between recreational PA (e.g., walking in a park) and everyday activities (e.g., commuting to work and shopping) using the accelerometer data we collected. Without a complete separation of these two types of PA, there may still be mis-specificity between the exposure metrics and the PA outcomes measured, as different environmental characteristics (e.g., parks, walkability) may be more likely to be associated with a particular type of PA outcome [24]. Third, our results are subject to the uncertain geographic context problem(UGCoP), which refers to the uncertainties of contextual exposures that influence health behaviors or outcomes [18]. Yet, to mitigate UGCoP, we reran the models with exposure metrics derived using 100-m buffer and another activity space method, and the results were relatively robust. Fourth, due to the cross-sectional nature of our analyses and the lack of information examining the decision-making processes of each PA scenario, our results may be subject to selective daily mobility bias [39]. This refers to the fact that we are not sure whether an exposure (e.g., visiting a park) leads to a PA outcome (e.g., a walk) or whether the exposure (e.g., the park visits) is in fact part of a pre-planned activity (e.g., a family picnic). As a result, our results are limited in their causal interpretation. Fifth, although our analytic sample of 350 person-days of environmental exposure and PA data allowed us to examine day-to-day relationships between environmental exposures and PA outcomes within an individual (i.e., whether higher or lower exposure on a given day is associated with the participant’s day-level PA outcomes), the relatively small person-level sample size (*N* = 55) limited our examination of between-person relationships (i.e., whether a participant's higher or lower average exposure across days is associated with their average PA outcomes). Sixth, we found that blue space water was also used in deriving the EPA layer of greenspace along walkable roads layer in the EnviroAtlas data, but given that we used existing data and the small number of water bodies in LA. We do not expect this to be a major source of noise in the metrics. Finally, we focused on a health-disadvantaged group of low-income Hispanic women, a population that has been poorly studied and is disproportionately exposed to various environmental hazards. Therefore, our results may not be generalizable to pregnant women in other regions or SES or racial/ethnic groups.

To our knowledge, this is the first study to examine GPS-based greenspace and walkability exposure and accelerometer-assessed PA in women during pregnancy and the early postpartum period. A major strength is the repeated collection of high-resolution smartphone location and accelerometer data in the first and third trimesters of pregnancy and 4–6 months postpartum. Consequently, we overcome recall errors associated with self-reported exposures and PA outcomes and provide insights into longitudinal changes in both exposures and outcomes. In addition, the study applies a GPS-based exposure assessment method to capture women’s exposures to greenspace and walkability in the daily activity space and examine the relationships between these exposures and pregnant women’s PA outcomes. As a result, our study should reduce spatial and temporal misclassification between environmental exposures and PA outcomes, a problem that has been prevalent in residential-based studies. Furthermore, the longitudinal design of this study allows us to examine both between-subject effects (variations in exposures between pregnant women) and within-subject effects (diurnal variations for each woman) of exposures on pregnant women’s PA outcomes, which was not possible in previous studies that examined exposures at one residence and at one time point late in pregnancy or around delivery.

## Conclusion

Pregnancy and early postpartum are critical periods for maternal and child health. Our findings that daily exposure to greenspace and a walkable environment is associated with more MVPA during this period can be used by landscape architects, urban planners, and policy makers to implement urban interventions to improve greenspace and neighborhood walkability, especially in low SES neighborhoods. Our findings could also provide further evidence for the formulation of effective environment-based strategies to promote PA among women across pregnancy and postpartum and reduce the short- and longer-term health risks and consequences associated with lack of PA during this period. Future studies are recommended to investigate how different aspects of parks and open space (amenities, accessibility, and maintenance) influence PA in pregnant women to further elucidate the behavioral mechanism between exposure to recreational spaces and maternal and infant health.

## Supplementary Information

Below is the link to the electronic supplementary material.

Supplementary file1 (DOCX 4205 KB)

## Data Availability

Access to MADRES data, which include the analytical datasets used in this study, requires the proposal and approval of the specific research project and is not directly sharable. Details on obtaining access are available at https://madres.usc.edu/.
