# Supplementary material for: Smartphone GPS-Based Exposure to Greenspace and Walkability and Accelerometer-Assessed Physical Activity During Pregnancy and Early Postpartum—Evidence from the MADRES Cohort
Source: J Urban Health. 2024 Aug 15;101(6):1128–42. doi: 10.1007/s11524-024-00903-6 (PMC11652437; doi:10.1007/s11524-024-00903-6)
Supplement: Supplementary file 1 — Supplementary file1 (DOCX 4205 KB) [file 11524_2024_903_MOESM1_ESM.docx]

**Supplementary Materials**

**Table S1.** Between- and within-subject effects of greenspace and walkability exposures derived using the 100 m KDE, 100 m route buffer, 800 m residential network buffer, and 1,600 m residential network buffer methods on daily MVPA outcomes

|  | **Daily MVPA minutes(log-transformed)** | | |  |
| --- | --- | --- | --- | --- |
|  | *100 m KDE model* | *250 route buffer model* | *800 m residential*  *network buffer model* | |
| *Predictors* | *Estimates (95%CI)^1^* | *Estimates (95%CI)^1^* | *Estimates (95%CI)^1^* | |
| % Green space along walkable roads (BS) | 1.00 (0.99 – 1.02) | 1.03 (1.00 – 1.06) | 1.01 (0.99 – 1.03) | |
| Distance to the nearest park entrance (BS) | 1.00 (0.97 – 1.04) | 1.04 (0.99 – 1.10) | 1.00 (1.00 – 1.00) | |
| Walkability index score (BS) | 1.03 (0.95 – 1.11) | 1.18 ^*^ (1.03 – 1.34) | 1.08 (0.99 – 1.18) | |
| Daily parks and open space exposure (BS) | 1.32 (0.80 – 2.19) | 1.46 (0.91 – 2.36) | 1.00 (0.75 – 1.33) | |
| % Green space along walkable roads (WS) | 0.99 (0.98 – 1.01) | 1.01 (0.99 – 1.03) |  | |
| Distance to the nearest park entrance (WS) | 0.99 (0.94 – 1.05) | 0.99 (0.96 – 1.02) |  | |
| Walkability index score (WS) | 1.00 (0.88 – 1.13) | 1.02 (0.94 – 1.12) |  | |
| Daily parks and open space exposure (WS) | 1.15 (0.97 – 1.36) | 1.22 ^*^ (1.02 – 1.47) |  | |
| Maternal age | 1.00 (0.97 – 1.02) | 0.99 (0.96 – 1.01) | 1.00 (0.98 – 1.02) | |
| Education: Some college/Graduate | 0.69 ^*^ (0.50 – 0.94) | 0.67 ^**^ (0.50 – 0.90) | 0.75 ^*^ (0.56 – 1.00) | |
| Parity: First-born | 1.11 (0.79 – 1.56) | 0.99 (0.72 – 1.37) | 1.07 (0.74 – 1.55) | |
| Employment status: Employed | 1.02 (0.82 – 1.27) | 1.02 (0.83 – 1.25) | 1.05 (0.85 – 1.30) | |
| Pre-pregnancy BMI category: Overweight/Obesity | 0.89 (0.64 – 1.22) | 0.95 (0.71 – 1.28) | 0.87 (0.64 – 1.18) | |
| Average daily temperature (°C) | 0.68 ^*^ (0.49 – 0.93) | 0.66 ^**^ (0.49 – 0.88) | 0.68 ^*^ (0.50 – 0.92) | |
| Type of day: Weekend | 1.02 ^*^ (1.00 – 1.04) | 1.02 ^*^ (1.00 – 1.04) | 1.02 ^*^ (1.00 – 1.04) | |
| The 3^rd^ trimester day | 0.81 ^**^ (0.71 – 0.93) | 0.81 ^**^ (0.70 – 0.92) | 0.80 ^**^ (0.70 – 0.92) | |
| 4-6 months postpartum day | 0.96 (0.81 – 1.14) | 0.95 (0.80 – 1.12) | 0.96 (0.81 – 1.14) | |
| Neighborhood cohesion and safety score (range from 1 to 5) | 0.90 (0.75 – 1.08) | 0.91 (0.76 – 1.09) | 0.90 (0.75 – 1.08) | |
| Daily accelerometry wearing hours | 1.04 (0.92 – 1.17) | 1.03 (0.92 – 1.15) | 1.02 (0.91 – 1.15) | |
| Total valid accelerometry collection days | 1.09 ^***^ (1.06 – 1.13) | 1.09 ^***^ (1.06 – 1.13) | 1.10 ^***^ (1.07 – 1.13) | |

^1^ Exponentiated effect estimates interpreting on a multiplicative scale were reported for all models.

^2^ The binary variable was not person-mean centered for the ease of interpretation. The person-mean centered versions of the variable were also tested, and results remained invariant.

**Table S2**. Effect estimates and 95% confidence intervals (CI) produced from simple slope analyses for significant interaction terms (p < .05)

| **Exposure x Effect Modifiers** | **Estimates (95%CI)** |
| --- | --- |
| **Activity space walkability x 4-6 months postpartum** | |
| 1^st^ and 3^rd^ trimester days | 1.12 (0.95-1.31) |
| The 4-6 months postpartum days | 0.81 (0.63-1.03) |
| **Activity space walkability x Pre-pregnancy BMI category** | |
| Pre-pregnancy BMI category: Overweight | 0.99 (0.77-1.27) |
| Pre-pregnancy BMI category: Obese | 1.26 (1.02-1.56)* |
| Pre-pregnancy BMI category: Normal | 0.85 (0.64-1.13) |
| **Activity space street %greenspace x Maternal parity** | |
| Maternal parity: 1st born | 1.02 (0.99-1.06) |
| Maternal parity: >=2nd born | 0.98 (0.96-1.01) |
| **Activity space park exposure x Neighborhood safety and cohesion** | |
| Neighborhood cohesion and safety: Low (-1SD) | 1.57 (1.2-2.04)*** |
| Neighborhood cohesion and safety: Medium (Mean) | 1.27 (1.05-1.52)* |
| Neighborhood cohesion and safety: High (+1SD) | 1.02 (0.81-1.3) |

** p<0.05   ** p<0.01   *** p<0.001*

*Notes.* BMI = body mass index.
